# The Dynamic Changes of DNA Methylation and Histone Modifications of Salt Responsive Transcription Factor Genes in Soybean

**DOI:** 10.1371/journal.pone.0041274

**Published:** 2012-07-18

**Authors:** Yuguang Song, Dandan Ji, Shuo Li, Peng Wang, Qiang Li, Fengning Xiang

**Affiliations:** The Key Laboratory of Plant Cell Engineering and Germplasm Innovation, School of Life Sciences, Shandong University, Jinan, Shandong, China; University of Georgia, United States of America

## Abstract

Epigenetic modification contributes to the regulation of gene expression and plant development under salinity stress. Here we describe the identification of 49 soybean transcription factors by microarray analysis as being inducible by salinity stress. A semi-quantitative RT-PCR-based expression assay confirmed the salinity stress inducibility of 45 of these 49 transcription factors, and showed that ten of them were up-regulated when seedlings were exposed to the demethylation agent 5-aza-2-deoxycytidine. Salinity stress was shown to affect the methylation status of four of these ten transcription factors (one *MYB*, one *b-ZIP* and two *AP2/DREB* family members) using a combination of bisulfite sequencing and DNA methylation-sensitive DNA gel blot analysis. ChIP analysis indicated that the activation of three of the four DNA methylated transcription factors was correlated with an increased level of histone H3K4 trimethylation and H3K9 acetylation, and/or a reduced level of H3K9 demethylation in various parts of the promoter or coding regions. Our results suggest a critical role for some transcription factors' activation/repression by DNA methylation and/or histone modifications in soybean tolerance to salinity stress.

## Introduction

Soybean (*Glycine max* (L). Merr.) is an important source of protein and oil in both the human and domestic animal diet. As for most crop species, its productivity is significantly compromised by soil salinity [1], but, like most plants, it has evolved a variety of mechanisms to aid its survival under environmental stress. The expression of many plant genes is altered by salinity stress; some of these encode aspects of cellular metabolism and stress tolerance, while others are regulatory in nature [1], [2]. Transcription factors (TFs), which belong to the latter class, have been classified into a number of families on the basis of their sequence, and some members of the *MYB*, *NAC*, *b-ZIP* and *AP2-DREB* families have been shown to be intimately involved in the stress response [3], [4], [5], [6], [7], [8]. Such as, the heterologous expression of three soybean *MYB* and three *b-ZIP* TFs in *Arabidopsis thaliana* improved its response to salinity and freezing stress [9], [10]. Similarly the heterologous expression of *GmDREB2* was able to enhance the drought and salinity tolerance of tobacco [11], as did the over-expression of either *GmNAC11* or *GmNAC20* for soybean [12].

Once a plant detects the onset of stress, TFs characteristically respond by inducing the expression of a cascade of downstream targets. However, their activation is in part also dependent on their chromatin structure, which is largely determined by epigenetic means [13], [14], [15], [16]. Cytosine methylation within the promoter sequence has been shown to underlie numerous cases of gene down-regulation or silencing [17], [18], [19], [20], [21]. DNA methylation in the plant genome mostly at CG dinucleotides and CNG trinucleotides, but also at an asymmetrical sequence contexts CNN (N is any nucleotide but G) [22], [23], [24]. The N terminus of the histone molecule can be acetylated, phosphorylated, methylated, ubiquitinated or ribosylated [25]. The presence of the trimethylated form of histone H3K4 and of the acetylated form of H3K9 in the promoter region have been frequently associated with transcriptional activation, while that of the dimethylated form of H3K9 represses it [26], [27], [28]. Sometimes, H3K9 methylation can trigger cytosine methylation in both *Neurospora crassa* [29] and *A. thaliana* [30], while cytosine methylation at the CNG trinucleotide appears to be partially dependent on the activity of a histone methyltransferase [29], [31], [32].

A number of examples where epigenetic modification has contributed to the regulation of gene expression during periods of environmental stress have been presented. In particular, the low temperature induced expression of the maize gene *ZmMI1* has been correlated with a reduction of DNA methylation in its nucleosome core [33]. In tobacco, several stress agents are known to promote demethylation in the *NtGPDL* coding sequence, leading to alterations in its level of expression [34]. The submergence of rice seedlings reduces histone H3K4 trimethylation and acetylation in genes encoding both alcohol dehydrogenase and pyruvate decarboxylase, leading to their up-regulation [35]. In *A. thaliana*, the drought-induced expression of a number of stress-responsive genes has been associated with an increase in H3K4 trimethylation and H3K9 acetylation [36]. Here, we set out to document the induction by salinity stress of DNA methylation and histone modification in a number of salinity responsive soybean TFs, and to identify what relationship there is, if any, between the expression of a TF and the epigenetic status of its promoter sequence. In addition, since to date no systematic attempt has been made to investigate the dynamics and reversibility of both DNA and histone modification over the course of a stress episode, we have explored this feature focusing on four salinity stress inducible soybean TFs.

**Figure 1:**
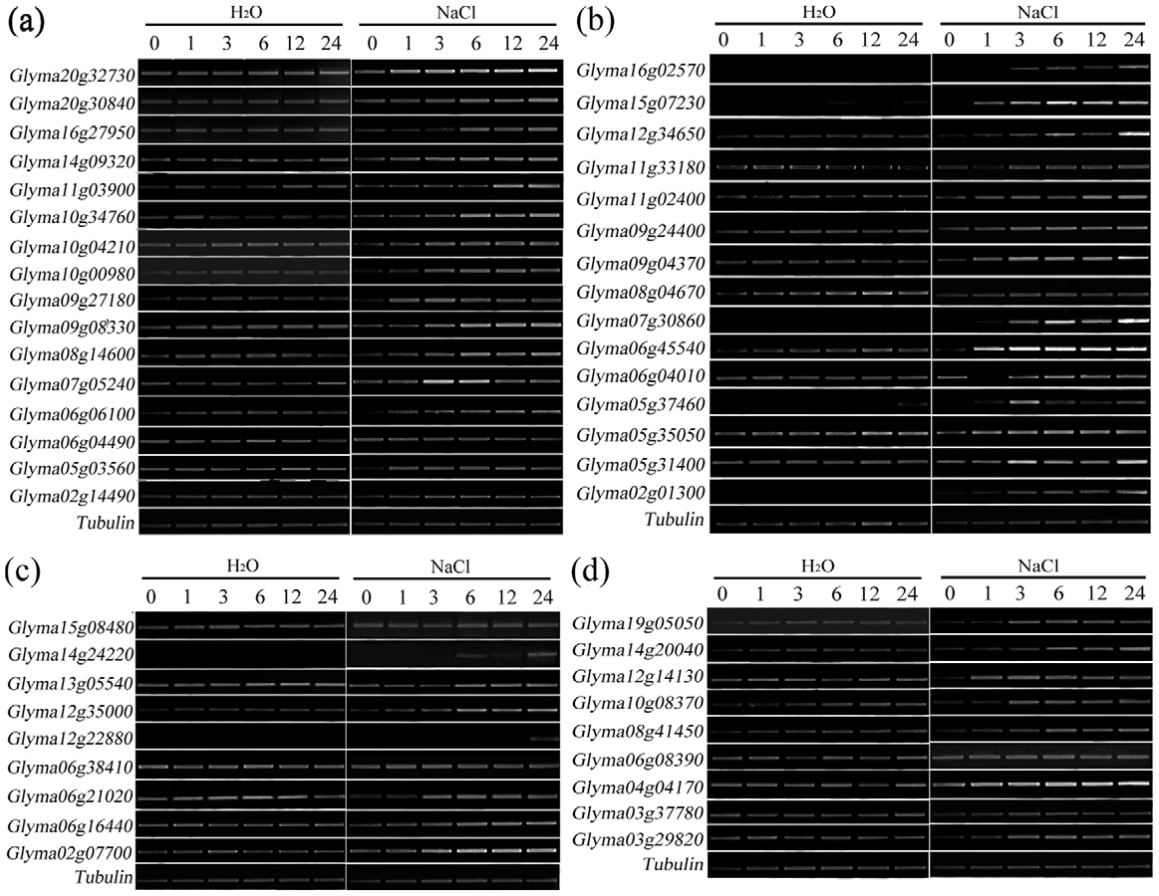
The expression of the 49 TFs in mock-stressed and salinity-stressed seedlings. (a) *GmAP2-DREBs*, (b) *GmMYBs*, (c) *GmNACs* and (d) *Gmb-ZIPs*. M0-M24 refer to seedlings exposed to just ddH_2_O for, respectively, 0h, 1h, 3h, 6h, 12h and 24h; S0–S24 refer seedlings exposed to 150 mM NaCl for 0h, 1h, 3h, 6h, 12h and 24h, respectively. Each gene-specific region was amplified by RT–PCR using the gene-specific primers (Table S2). The *TUBULIN* gene (Genbank accession AY907703) was used as an internal control. The experiment was repeated three times with similar result.

## Materials and Methods

### Exposure of soybean seedlings to salinity stress and 5-ADC treatment

Seedlings of the soybean cultivar Williams 82 were grown in vermiculite under a 16h photoperiod at 25°C for 14 days before being exposed to stress treatment. Once the seedlings had been removed from the vermiculite and their roots rinsed in water, they were then treated with either 150 mM NaCl for 1h, 3h, 6h, 12h or 24h, or with 50 µM 5-aza-2-deoxycytidine (5-ADC) for 12h, 24h, 48h or 72h. RNA and DNA was extracted from snap-frozen plants both before the stress treatment had begun and then at each time interval. Mock treatments (ddH_2_O only) were included as a control. RNA was prepared from 0.2 g plant material using the TRIzol (Invitrogen) reagent, following the manufacturer's protocol, and DNA was extracted from 1 g plant material using a DNeasy Plant Mini kit (Qiagen).

**Figure 2:**
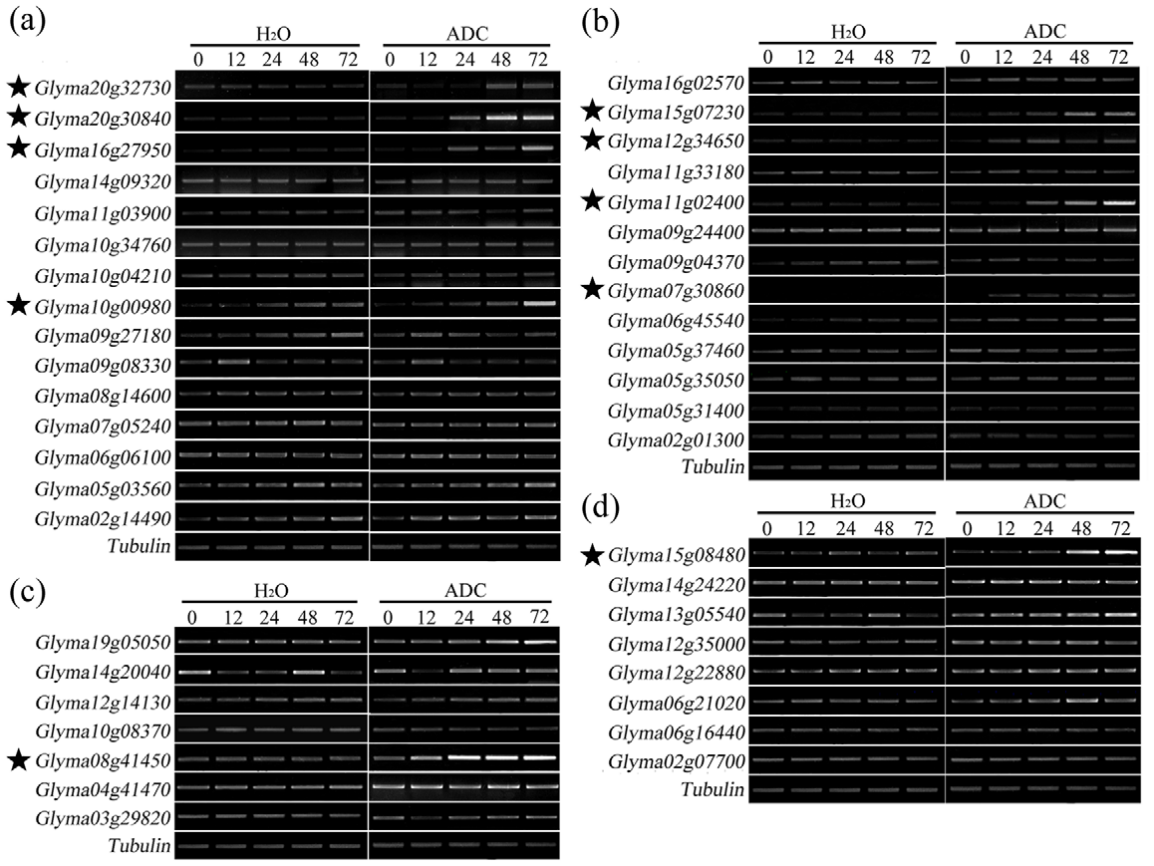
Expression of 45 salinity inducible TFs in seedlings exposed to 5-ADC treatment. (a) *GmAP2-DREBs*, (b) *GmMYBs*, (c). *GmNACs*. and (d) *Gmb-ZIPs*. M0-M72 refers to seedlings treated with water only for, respectively 0h, 12h, 24h, 48h and 72h, while A0-A72 refer to seedlings exposed to 50 µM 5-ADC for 0h, 12h, 24h, 48h and 72h, respectively. Each gene-specific region was amplified by RT–PCR using the gene-specific primers (Table S2). The *TUBULIN* gene (Genbank accession AY907703) was used as an internal control. The experiment was repeated three times with similar result.

### Microarray analysis

RNA were isolated from the mock (M0, M1, M3, M6, M12, M24) and salinity treated (S0, S1, S3, S6, S12, S24) seedlings. 0.5 µg RNA that extracted from each time point of the mock and salinity-stressed seedlings were mixed respectively to obtain the mock and salinity-stressed RNA pools, and then they were used to synthesize the cDNA. The cDNA was labeled with biotin, and then hybridized to an Affymetrix soybean Genome Array according to the manufacturer's instructions (15h in a rotating hybridization oven set at 45°C and 60 rpm). After the hybridization, the microarrays were scanned using a GeneChip® Scanner 3000 (Affymetrix, P/N 00-00212). Then the scaling factor, background, noise, and percentage presence were calculated according to the Affymetrix Data Mining Tool protocols (Affymetrix). All resulting datasets were filtered using the absolute call metric (present or absent) implemented within Microsoft Access (Microsoft Corporation, Redmond, WA) and the microarray data were processed in an R (v2.7.0) environment, using the LIMMA package [37]. Quantile normalization was performed. A single repeat microarray analysis for each group was performed.

**Figure 3:**
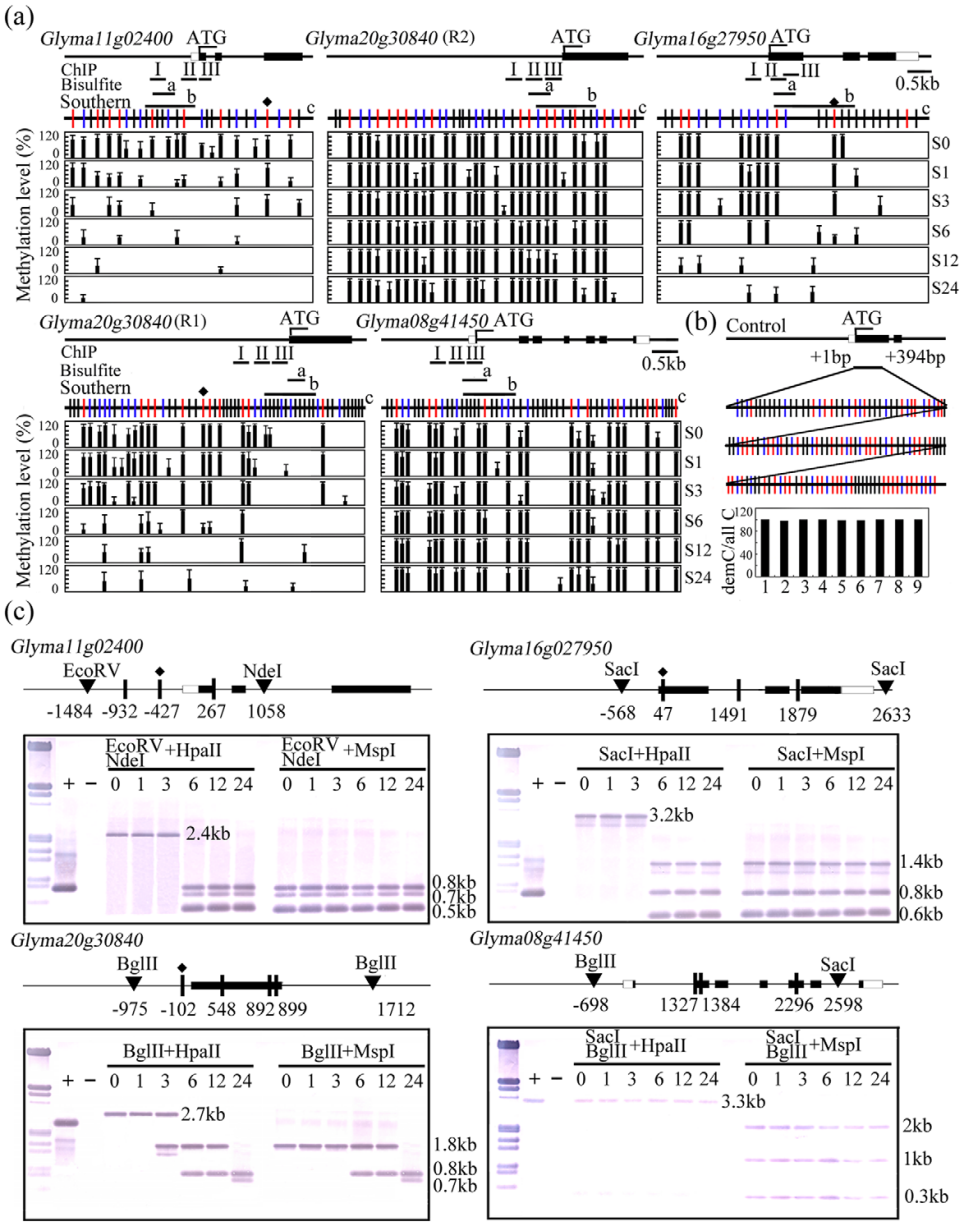
Methylation status of the promoter region of four salinity-responsive TFs in untreated (S0) and salinity-stressed (S1–S24) seedlings (S1: 1h, S3: 3h, S6: 6h, S12: 12h, S24: 24h). (a) The black and white boxes indicate, respectively, exon and untranslated regions. The short bars annotated with “I, II, III”, “a” or b'' indicate, respectively the sequences subjected to ChIP analysis, genomic bisulfite sequencing and those used as probes for Southern blotting. The long vertical bars marked “c” display the distribution of CG dinucleotides (marked with red vertical lines), and CNG (blue vertical lines) and CNN (black vertical lines) trinucleotides. The red vertical lines marked with a rectangular indicate CCGG sites analyzed by Southern blotting. The thick black vertical lines represent the proportion of methylated cytosine. Ten positive clones from each gene's amplicon were sequenced. The data reflect the outcome of three independent experiments, and error bars represent standard error (SD). (b) The efficiency of the bisulfite treatment to transform unmethylated cytosine to thymine. A fragment of *Glyma20g32730* with numerous cytosines was cloned into Dm- *E. coli* cells and the plasmid was treated with bisulfite in parallel with the soybean genomic DNA. All clones processed showed a transformation rate >99.7%. (c) Methylation-sensitive DNA gel blot analysis of non-stressed (S0) and salinity-stressed seedlings (S1–S24). Genomic DNA was digested to generate large fragments, then with one or other of the schizomers *Hpa*II or *Msp*I. Hybridization probes indicated. A DNA fragment amplified from the probe sequence was used as a positive control (+), and ddH_2_O was used as a negative control (−).

### Transcript level analysis

Semi-quantitative RT-PCR (sqRT-PCR) and quantitative real-time RT-PCR (qRT-PCR) were employed to quantify transcript levels more precisely. RNA was extracted from 0.2 g of seedling material ground in liquid nitrogen by the addition of 1 ml TRIZOL reagent (Invitrogen) and treated with RNase-free DNase I. A 3 µg aliquot of total RNA was used to generate the first cDNA strand with the SuperScript First-Strand Synthesis System (Invitrogen) according to the manufactuter's instructions. A 1 μl aliquot of this cDNA was used as the template for a 22–34 cycle sqRT-PCR, where the cycling regime was 94°C/30 s, 55°C/30 s, 72°C/30 s. A fragment of the soybean *TUBULIN* gene (Genbank accession AY907703) was used as a reference. Primer sequences are given in Table S2. Each 15 μl qRT-PCR contained 7.5 µl Maxima SYBR Green qPCR Master mix buffer (Roche), 0.5 µl 10 µM specific primers, 1.5 µl of a 1∶10 dilution of cDNA and 5.5 µl ddH_2_O. The cycling regime consisted of a denaturation step (95°C/3 min) followed by 18–35 cycles of 95°C/30 s, 60°C/15 s, 72°C/15 s, and a fragment of the soybean *TUBULIN* gene (GenBank accession AY907703) was used as a internal control. Primer sequences are given in Table S2. The relative expression level of the target sequence was determined using the 2^−ΔΔCt^ method [38]. Each estimate was derived from the mean of three independent biological replicates.

**Figure 4:**
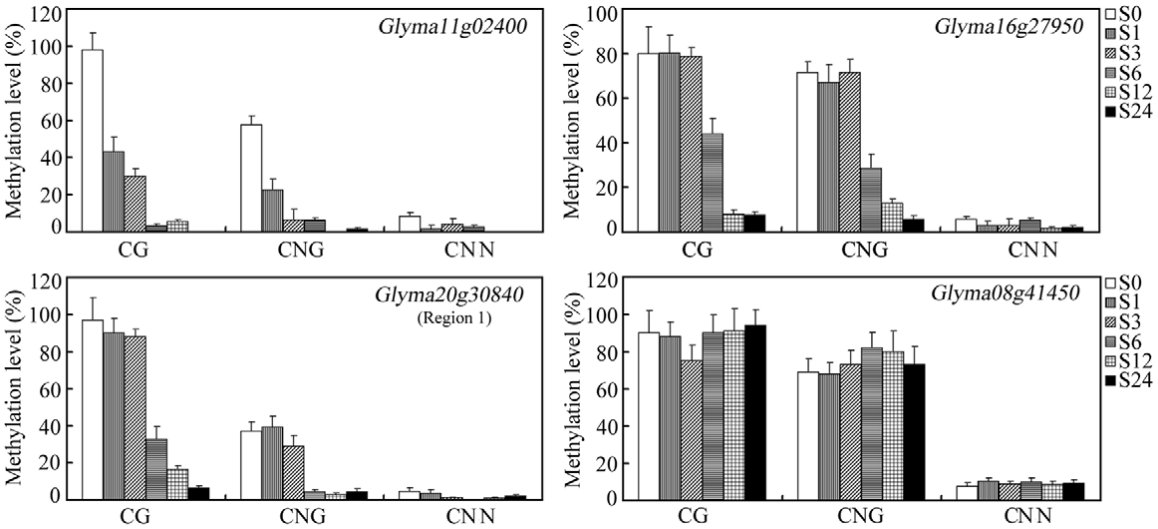
DNA methylation patterns in *Glyma11g02400*, *Glyma16g27950*, *Glyma08g41450* and *Glyma20g30840* in non-stressed (S0) and salinity-stressed (S1 S24) seedlings. The left axis shows the percentage of methylated cytosines at each site (present as CG, CNG and CNN). The data represent the mean of three biological replicates. Error bars represent standard errors.

### Bisulfite DNA sequencing

A 2 μg DNA aliquot was dissolved in 50 μl ddH_2_O and denatured by adding 5.5 μl 3 M NaOH and incubating for 30 min at 42°C. Thereafter, 510 μl 2.3 M sodium bisulfite (pH 5.0), 30 μl 10 mM hydroquinone and 65 μl ddH_2_O were added, and the solution overlaid with mineral oil and held for 16h at 55°C. The DNA was recovered using a Wizard® DNA Clean-Up System kit (Promega A7280), and a 90 μl aliquot treated with 10 μl 3 M NaOH for 15 min at 37°C, then neutralized by adding 70 μl 10 M ammonium acetate. Finally, the DNA was precipitated by adding 400 μl ethanol and 10 μl glycogen, and re-suspended in 50 μl ddH_2_O to provide the template for a series of PCRs based on the gene-specific primers listed in Table S2. A fragment of *Glyma20g32730* featuring many CG, CNG and CNN sites was amplified from genomic DNA, then inserted into *pMD18-T* vector and transferred into Dm- *E. coli* strain JM110. The plasmid was released from the bacterial cells by the plasmid extraction kit (TianGen. Cat. DP103-03) and treated with bisulfite in parallel with the soybean genomic DNA as a control to monitor the transformation efficiency of unmethylated cytosine to thymine. The subsequent PCR consisted of 34–37 cycles of 94°C/30 s, 55°C/30 s, and 72°C/40 s. The resulting amplicons were purified with a Wizard® DNA Clean-Up System kit, ligated into the *pMD18-T* vector (TaKaRa) and transferred into *E. coli* for sequencing. Ten clones from each amplicon were sequenced. The process was repeated three times using biologically independent samples.

**Figure 5:**
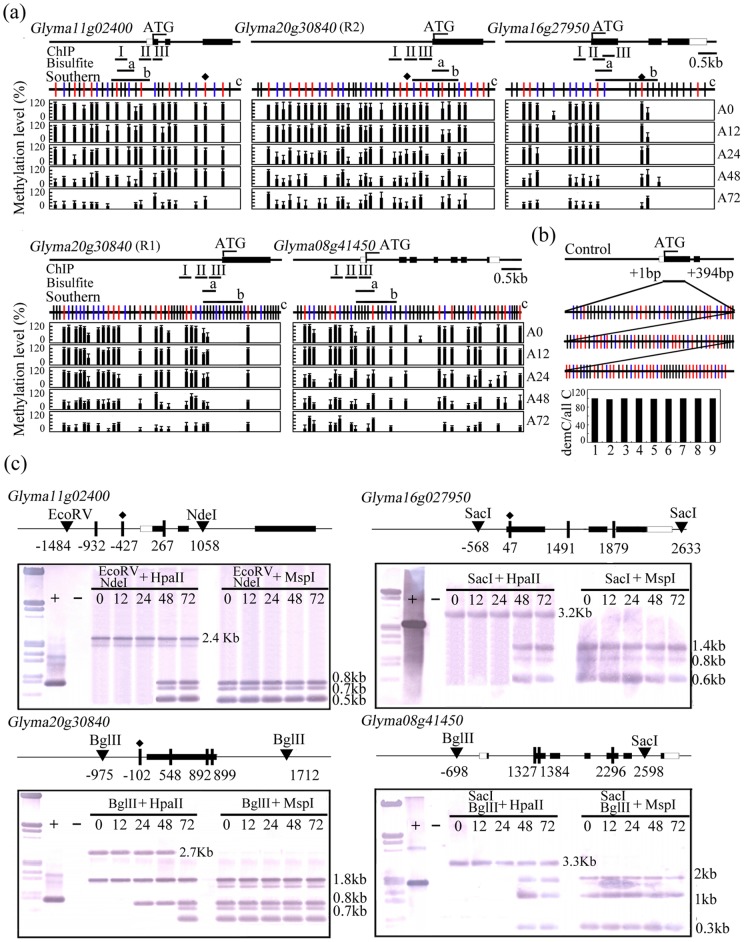
Promoter methylation status in four salinity-responsive TFs in non-treated (A0) and 5-ADC treated seedlings (A12–A72). For Figure legend please refer to Figure 3 legend.

### Chromatin immunoprecipitation (ChIP) assay

The ChIP protocol was modified from that of Johnson *et al*. (2002). Briefly, 1 g of plant tissue was fixed by immersion in 1% v/v formaldehyde under vacuum for 10 min. The extracted DNA/protein complex was then sheared by sonication to a size range of ∼100–1000 bp. After centrifugation, the complex was immunoprecipitated by challenging with H3K9ac, H3K4me3 and H3K9me2 antibodies (Millipore cat. 07–392, 07–473 and 05–768R) at a titer of 1∶100. The residual protein was degraded by the addition of 10 μl (20 mg/ml) proteinase K, followed by a phenol/chloroform extraction. A 2 μl aliquot of the final solution was used as a template for qRT-PCR analysis as described above. A 1000x diluted input DNA (Input) obtained from 500 μl of extract was purified in parallel with the immunoprecipitated samples as a control, and ChIP reactions were also performed in the absence of antibody (No AB) to detect the occurrence of any non-specific binding. Relative levels of H3K9 acetylation, H3K9 dimethylation and H3K4 trimethylation were normalized to an internal control (GenBank accession AY907703). The sequences of all PCR primers used are given in Table S2. The mean and standard deviation are shown for three independent ChIP experiments and the significance of differences between means assessed with a *t* test.

**Figure 6:**
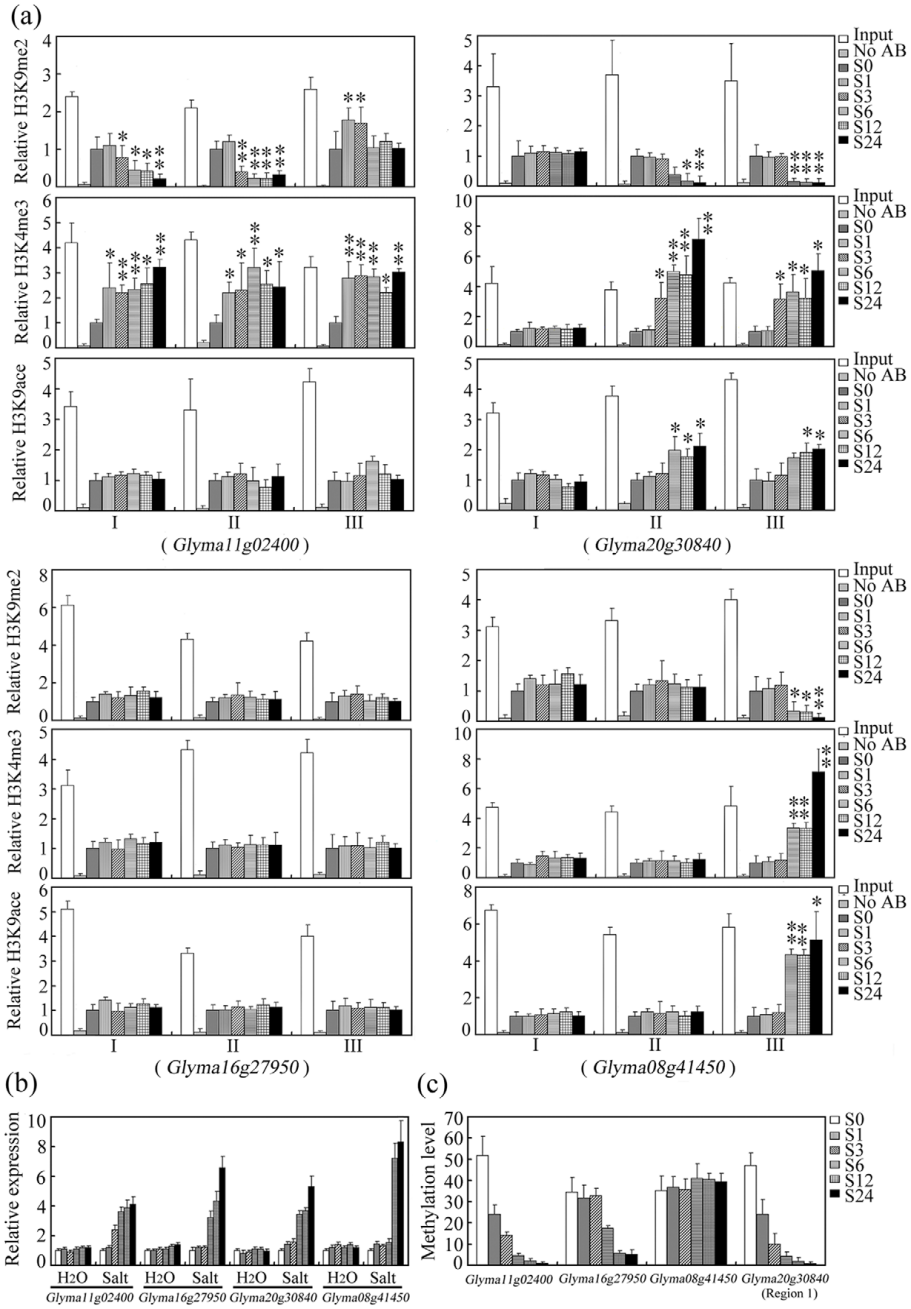
Expression, DNA methylation and histone modification status of *Glyma11g02400, Glyma16g27950, Glyma08g41450* and *Glyma20g30840* in none treated (S0) and salinity-stressed (S1–S24) seedlings. (a) Relative H3K9 demethylation, acetylation and H3K4 trimethylation content (ChIP assay). A 1∶1,000 dilution of input DNA (Input) served as a control for PCR amplifications and the ChIP reactions carried out in the absence of antibody (N0 AB). Relative H3K9 acetylation, H3K9 dimethylation and H3K4 trimethylation were determined by qRT-PCR and normalized to an internal control *TUBULIN* gene (Genbank accession AY907703). Data represent the mean of three biological replicates. Asterisks indicate means differing significantly from the S0 situation. Error bars represent standard errors. *P<0.05, **P<0.01. (b) Gene expression (qRT-PCR) profiles. (c) Cytosine methylation level (bisulfite sequencing).

### Methylation-sensitive Southern blot analysis

Genomic DNA (100 µg) extracted from both non-stressed and salinity-stressed seedlings was treated for at least 6h with 100 U of the appropriate restriction enzymes (*EcoR*V and *Nde*I for *Glyma11g02400*; *Sac*I for *Glyma16g27950*; *Bgl*II for *Glyma20g30840*; *Sac*I and *Bgl*II for *Glyma08g41450*) (TaKaRa) to generate large fragments containing the target sequences. The digested DNA was extracted by phenol/chloroform and divided into two equal aliquots, one of which was treated with *Hpa*II and the other with its schizomer *Msp*I [39]. The digested DNA was re-extracted, electrophoretically separated through an 0.8% agarose gel and transferred onto a Hybond N^+^ membrane (Amersham). Probes for each gene were designed to detect the methylation status within the target sequence that analysed by genomic bisulfite sequencing. About 3 μg of probe DNA was labeled using a DIG-High Prime kit (Roche), and the subsequent hybridization and detection procedure was performed using a DIG High Prime DNA Labeling and Detection Starter Kit I (Roche), according to the manufacturer's instructions. The positive control consisted of a 100x diluted DNA fragment that amplified from the genomic DNA in the same regions that prepared for probes, while the negative control was ddH_2_O.

## Results

### The identification of salinity stress responsive TFs in soybean

A set of differentially expressed genes were identified by comparing the soybean Affymetrix microarray profiles generated by probing with RNA extracted from salinity-stressed and non-stressed plants. We mainly focused on the four groups of *AP2/EREB, bZIP, NAC* and *MYB* transcription factors that have been verified for salt stress in *Arabidopsis* or other plants. Of the 1,335 *MYB, NAC, AP2/DREB* and *b-ZIP* TFs represented on the microarray, 49 appeared to be up-regulated (fold change of hybridization signal >2, *p*<0.01) by salinity stress. These consisted of 15 (of 448) *GmMYB*s, 9 (of 226) *GmNAC*s, 16 (of 426) *GmAP2/DREB*s and 9 (of 235) *Gmb-ZIP*s (Table S1). When the expression of these 49 TFs was assayed by sqRT-PCR in both mock-stressed (M0–M24) and salinity-stressed soybean seedlings (S1–S24) with gene-specific primers (Table S2), 14 of the *GmMYBs*, 8 of the *GmNACs*, 15 of the *GmAP2/DREBs* and 8 of the *Gmb-ZIPs* were confirmed to be markedly induced by salinity stress (Figure 1). Expression pattern analysis indicated that 19 of them were strongly induced at a relatively early stage of exposure to salinity (1–3h), while the others were induced somewhat later (6–24h).

### Expression of the salinity induced TFs in the presence of 5-ADC

The expression of the 45 salinity-induced TFs was then monitored in the mock treated (M0–M72) and the seedlings that exposed to 5-ADC for various periods (A0–A72). As a result, ten of the them showed higher levels of expression in treated (M0–M72) than in mock-treated (A0–A72) seedlings; these ten TFs consisted of four *GmMYB*s (*Glyma11g02400*, *Glyma07g30860*, *Glyma12g34650, Glyma15g07230*), one *GmNAC* (*Glyma15g08480*), four *GmAP2/DREB*s (*Glyma20g32730*, *Glyma20g30840*, *Glyma16g27950*, *Glyma10g00980*) and one *Gmb-ZIP* genes (*Glyma8g41450*) (Figure 2). The expression level of nine of these TFs was very low for the first 12h of exposure, but thereafter rose substantially; the exception was the *Gmb-ZIP Glyma08g41450*, the expression of which was induced somewhat earlier.

### Methylation status as affected by salinity stress

To investigate the DNA methylation status of above candidate genes under salinity stress, the sequence corresponding to the translation start codon and the promoter region of the ten TFs was subjected to bisulfite sequencing. First, the efficiency of the sodium bisulfite treatment to convert cytosine to thymine was estimated. The efficiency of the sodium bisulfite treatment to convert cytosine to thymine in the cytosine rich segment of *Glyma20g32730* was estimated to be 99.7%. Bisulfite sequencing result indicated that the *Glyma11g02400, Glyma08g41450, Glyma16g27950* and *Glyma20g30840* promoters all appeared to be differentially methylated by the imposition of salinity stress (Figure 3a, b, Figure S1), but those of the other six genes were largely non-methylated (Figure S2). In the *Glyma11g02400* promoter from position −518 to −274, most of the cytosines were demethylated following exposure to salinity stress for 1–24h (Figure 3a). In the immediate downstream region of the *Glyma16g27950* transcription start codon (+24 to +233), about 35% of the cytosines were methylated both before the salinity stress was imposed and for the first three hours of stress, but thereafter only few methylated cytosines remained (Figure 3a). In the *Glyma20g30840* promoter region 1 (−87 to +163), 51% of the cytosines were methylated prior to exposure to salinity stress, but this proportion fell to 27% after 1h, 12% after 3h, and to even lower levels as the stress was prolonged further (Figure 3a); meanwhile in region 2 of the same promoter (−163 to −405), 42% of the cytosines were methylated at the start of the stress period, and this proportion hardly altered thereafter (Figure 3a). In the *Glyma08g41450* region immediately downstream of the transcription start codon (+24 to +233), 35% of the cytosines were methylated prior to the imposition of stress, and the same proportion was maintained throughout (Figure 3a).

DNA methylation-sensitive Southern blotting was applied to verify these observations. The restriction fragments including *Glyma11g02400*, *Glyma16g27950* and *Glyma20g30840* were more readily digested by *Hpa*II after 6h of salinity stress than the same samples obtained from non-stressed seedlings, which consistent with a reduction in global cytosine methylation caused by salinity stress analyzed by bisulfite sequencing (Figure 3a, c). The sequence surrounding *Glyma08g41450* was not digestible by *Hpa*II, but several small restriction fragments were generated by *Msp*I digestion, suggesting that CCGG sites in the region of this TF were hypermethylated in both non-stressed and stressed seedlings (Figure 3c). Thus the Southern blotting outcomes were in general consistent with the bisulfite sequence data.

The analysis of DNA methylation pattern of them indicated that methylation was affecting either CG dinucleotides or CNG/CNN trinucleotides under salinity stressed process (Figure 4). In the *Glyma11g02400* promoter, 98% of the CG's, 60% of the CNG's and 6% of the CNN's were methylated in the non-stressed seedlings, but almost all the CG's, CNG's and CNN's were demethylated in plants exposed to salinity stress for more than 6h (Figure 4). For *Glyma16g27950*, some 80% of the CG's, 72% of the CNG's and 4% of the CNN's were methylated in non-stressed seedlings and those during the early phase (1–3h) of the salinity treatment, but by 6h a significant fall in CG and CNG methylation was observed (Figure 4). Within region 1 of the *Glyma20g30840* promoter, 95% of the CG's, along with 40% of the CNG's and 4% of the CNN's, were methylated at 3h after the imposition of stress, but by 6h, a marked reduction in CG and CNG methylation had occurred. CG, CNG and CNN methylation in the *Glyma08g41450* promoter was unaffected by salinity stress (Figure 4). Clearly, DNA methylation in *Glyma11g02400, Glyma16g27950* and *Glyma20g30840* (region 1) varied over the period of the salinity stress episode.

### Methylation status of Glyma11g02400, Glyma08g41450, Glyma16g27950 and Glyma20g30840 as affected by the presence of 5-ADC

To identify whether the up-regulation of these four genes were related with cytosine demethylation under 5-ADC treatment, the effect on the DNA methylation status of the four responsive TFs in plants treated with 5-ADC was analyzed using genomic bisulfite sequencing. The relevant test for the transformation efficiency of unmethylated cytosine to thymine using Glyma*20g32730* is illustrated. As a result, all four TFs were hypermethylated in non-treated seedlings; after a 24h exposure to 5-ADC, some evidence of demethylation was obtained, but from 48h onwards it was clear that a substantial level of demethylation had occurred (Figure 5a, b). This observation was supported by a DNA methylation-sensitive DNA gel blot (Figure 5c). All of the four TFs showed an increased digestion with HpaII after salinity stress for more than 48h, suggesting a reduction of cytosine methylation in 5-ADC stressed seedlings (Figure 5c).

### Histone modification of hypermethylated genes induced by salinity stress

The histone content (H3K4me3, H3K9ace and the inactive H3K9me2) of the four TFs (*Glyma11g02400*, *Glyma08g41450, Glyma16g27950* and *Glyma20g30840*) which responded to salinity stress by altering their methylation status was then examined, using a combination of ChIP and qRT-PCR (Figure 6a). An unmethylated gene *Glyma20g32730* was also analysed in parralle with them as a control (Figure S3). When *Glyma11g02400* was induced by salinity stress, a significant increase in H3K4me3 (regions I, II and III) and a decrease in H3K9me2 (regions I and II) was observed, but the H3K9ace sites remained unmodified (Figure 6a). Within *Glyma20g30840* (regions II and III) and *Glyma08g41450* (region III), a high level of H3K9me2 and a low level of H3K4me3 and H3K9ac was present in both non-stressed seedlings and those sampled during the early phase (1–3h) of salinity stress; at later time points (6–24h), a significant decrease in H3K9me2 and increase of H3K4me3 and H3K9ac content was observed (Figure 6a). A similar H3K4me3, H3K9ac or H3K9me2 signal was detected in all three regions of the *Glyma16g27950* promoter (Figure 6a). Thus, like the DNA methylation, histone modification was also subject to dynamic change during the course of the salinity stress episode.

### Changes of epigenetic modification in regulating the TFs expression during salinity stress

The expression of *Glyma11g02400* was low in non-stressed seedlings, while its promoter was hypermethylated and was highly enriched for H3K9me2 and depleted for H3K4me3 (Figure 6a, b, c). When the seedlings were exposed to salinity stress, its expression rose rapidly, while its promoter became gradually demethylated, the level of H3K9me2 fell and that of H3K4me3 increased (Figure 6a, b, c). *Glyma20g30840* behaved similarly, but the establishment of H3K4me3, DNA demethylation and gene expression were not contemporaneous. Demethylation was noticeable within 1h of the imposition of salinity stress, but the establishment of H3K4me3 did not occur until 3h and the up-regulation of expression only at 6h (Figure 6a, b, c). Similarly, *Glyma08g41450* was up-regulated by 12h after the start of the stress episode, but its promoter was hypermethylated throughout. Between 6h and 24h after the stress had begun, the level of H3K9me2 fell and that of H3K4me3 and H3K9ac rose (Figure 6a, b, c). *Glyma16g27950* expression was repressed in non-stressed seedlings and during the early phase of the stress episode, when its promoter was hypermethylated. Later the TF was gradually induced and its promoter demethylated, while the level of enrichment of H3K9me2, H3K4me3 and H3K9ac did not change. A correlation analysis of their expression, methylation levels and histone modifications indicated that up-regulation of *Glyma11g02400* was associated with a decreased level of DNA methylation (r = −0.89), H3K9me2 (r = −0.965, on average of regions II and III) and an increased level of H3K4me3 (r = 0.7, on average of regions I and II) (Figure 6; Table S3). The expression of *Glyma20g30840* correlated negatively with DNA methylaion (r = −0.78), H3K9me2 (r = −0.93 on average of regions II and III) and positively with H3K9ac (r = 0.96) and H3K4me3 (r = 0.93 on average) (Figure 6; Table S3). Up-regulation of *Glyma16g27950* just correlated negatively with DNA methylation (r = −0.97), did not with histone modifications, while the up-regulation of *Glyma08g41450* correlated negatively with H3K9me2 (r = −0.67) and positively with H3K9ac (r = 0.84) and H3K4me3 (r = 0.78), did not with DNA methylation during salinity stress (Figure 6; Table S3). Therefore, the histone accumulation/depletion and/or cytosine methylation appears to underlie the activation by salinity stress of these four TFs.

## Discussion

### TF transcription can be influenced by both DNA methylation and/or histone modification in a region specific manner

The regulation of genes via cytosine methylation and histone modification is a well recognized component of the plant stress response [14], [34], [40], [41]. Both these epigenetic modifications are region-specific, and can be dynamic over time [42], [43], [44], [45]. Here we have identified a set of ten salinity-induced, cytosine methylation-dependent TFs, three of which displayed the expected relationship between promoter cytosine methylation and gene expression during the salinity stress process, but one of which (*Glyma08g41450*) remained up-regulated even though it was in a highly methylated state (Figure 1, Figure 3a). A similar unexpected relationship has been noted for an embryogenesis-related gene in carrot [42]. Many genes are expressed despite their promoter region being highly methylated, so it seems probable that for *Glyma08g41450*, its up-regulation in plants exposed to salinity stress is independent of DNA methylation. One region of the *Glyma20g30840* promoter was hypermethylated in both stressed and non-stressed plants, while its neighbouring region responded to the stress by a reduction in methylation (Figure 3a). This behaviour provides an example of region-specific regulation of methylation. The gradual up-regulation of *Glyma11g02400* in salinity-stressed seedlings was accompanied by a decrease in CG, CNG and CNN methylation (Figure 4), while the rapid up-regulation of *Glyma20g30840* and *Glyma16g27950* was accompanied by a decrease in only CG and CNG methylation (Figure 4), suggesting heterogeneity in the genome for gene expression regulation via DNA methylation.

Histone modification provides a second major mechanism of epigenetic control over gene expression [46]. In a range of *A. thaliana* stress-responsive genes, salinity stress has been shown to increase trimethylation at H3K4 and decrease H3K9 demethylation [28]. The up-regulation of *Glyma20g30840* and *Glyma08g41450* during the course of the salinity stress episode may have been achieved by the depletion of H3K9me2 and the enrichment of H3K4me3 and H3K9ac in regions II and III of *Glyma20g30840* and in region III of *Glyma08g41450* (Figure 6a, b). The up-regulation of *Glyma11g02400* may have been brought about by the depletion of H3K9me2 and the enrichment of H3K4me3 in regions I, II and III (Figure 6a, b). Thus, as for DNA methylation, the effect of salinity stress on histone modification appears to be heterogeneous across the genome.

### Transcriptional activation, DNA methylation and histone modification are not simultaneous events

There was a distinct time lag between transcriptional activation, DNA methylation and/or histone modification of *Glyma11g02400*, *Glyma20g30840* and *Glyma08g41450*. Thus, most of the cytosine content of the *Glyma11g02400* promoter was demethylated very soon (within 1h) of the imposition of stress, but there was no evidence for the TF's activation before 3h (Figure 6b, c). Similarly, *Glyma20g30840* was progressively demethylated over the full 24h of the stress episode, but its up-regulation was complete within 6h (Figure 6b, c). In *Glyma11g02400*, *Glyma20g30840* and *Glym08g41450*, the H3K4me3 content was already increasing by 1h, but the up-regulation of TF expression occurred substantially later (Figure 6a, b). Similar time lags have been noted for the build-up of H3K4me3 within the coding regions of the *A. thaliana* drought-related genes *RD29A* and *RAP2.4* during drought stress [36]. Furthern more, the DNA demethylation of *Glyma20g30840* was prior to the erasure or establishment of H3K9me2, H3K4me3 and H3K9ac (Figure 6a, c) and all the three genes (*Glyma11g02400, Glyma20g30840* and *Glym 08g41450*) show an ealier establishment of H3K4me3 than H3K9me2 and H3K9ac (Figure 6a). Suggesting that there were also a time lage between the DNA demethylation and establishment of histone modifacitions.

### The interplay between DNA methylation and histone modification in the context of patterns of gene expression

In *A. thaliana*, it has been demonstrated that the loss of CG methylation in *met1* plants has a large effect on H3K9me2 content, leading to the idea that cytosine methylation can influence H3K9 modification [17], [47], [48], [49]. H3K9me2, mediated by KYP/SUVH4 and SUVH2, is also known to direct non-CG methylation [39], [50]. In the ‘two-step’ hypothesis for the regulation of transcription, CG methylation directs H3K9 methylation and H3K9 methylation recruits non-CG methylation [51]. Moreover, hypermethylation of the stress inducible genes in Arabidopsis correlated with the enrichment of H3K9me2 and depletion of H3K9ac histones under salt stress conditions [16]. In this study, the behaviour of *Glyma11g02400* and *Glyma20g30840* was consistent with this model; as CG was progressively demethylated in these TFs, the content of H3K9me2, H3K4me3 and/or H3K9ac rose and meanwhile a lower level of non-CG methylation was observed (Figure 6a, Figure 4). Note, however, that for *Glyma16g27950*, while salinity stress led to a noticeable demethylation of the DNA, it had little effect on the level of histone modification (Figure 6a, c). The *Glyma08g41450* promoter was hypermethylated throughout the salinity stress period, while H3K9me2 was depleted and H3K4me3 and H3K9ac accumulated (Figure 6a, c). For these two TFs, therefore, the evidence is that DNA methylation had no influence on histone modification, consistent with the behaviour of the *A. thaliana* genes *TOUSLED* and *RPA2* [52], [53].

## Supporting Information

Figure S1 **Monitoring the non-methylated cytosine to thymine transformation efficiency of the bisulfite sequencing procedure.** A fragment of *Glyma20g32730* with numerous cytosines was cloned into Dm- *E. coli* cells and the plasmid was treated with bisulfite. Nine positive clones of this gene from the bisulfite treated DNA were sequenced and compared with the untreated DNA sequence.
(PDF)

Figure S2 **Genomic bisulphite sequencing of six unmethylated genes in plants not exposed to salinity (S0) and those stressed for 1–24 h (S1–S24).** Ten clones of each gene were sequenced. The short thick bars indicate the regions subjected to sequencing, and the long vertical ones show the distribution of CG dinucleotides (black circles), and CNG (triangles) and CNN (unmarked) trinucleotides. The black vertical line represents the proportion of methylated sites. Data represent the mean of three biological replicates.
(PDF)

Figure S3 **ChIP analysis to assess the unmethylated gene** ***Glyma20g32730*** **'s H3K9me2, H3K9ac and H3K4me3 content in plants challenged with salinity.** The short bars marked “a” indicate regions subjected to genomic bisulfite sequencing (+1 to +393); “I, II and III” indicate the regions subjected to ChIP analysis.
(PDF)

Table S1 **The set of TFs identified by microarray analysis to be inducible by salinity stress.**
(DOC)

Table S2 **Sequences of primers employed in this research.**
(DOC)

Table S3 **Correlation analysis among methylation levels, gene expression and histone modifications of the four TFs during salinity stress. NC: no correlation;** *P **<0.05,** **P **<0.01.**
(DOC)
