# Supplementary material for: The Dynamic Changes of DNA Methylation and Histone Modifications of Salt Responsive Transcription Factor Genes in Soybean
Source: PLoS One. 2012 Jul 18;7(7):e41274. doi: 10.1371/journal.pone.0041274 (PMC3399865; doi:10.1371/journal.pone.0041274)
Supplement: Figure S2 — Genomic bisulphite sequencing of six unmethylated genes in plants not exposed to salinity (S0) and those stressed for 1–24 h (S1–S24). Ten clones of each gene were sequenced. The short thick bars indicate the regions subjected to sequencing, and the long vertical ones show the distribution of CG dinucleotides (black circles), and CNG (triangles) and CNN (unmarked) trinucleotides. The black vertical line represents the proportion of methylated sites. Data represent the mean of three biological replicates. (PDF) [file pone.0041274.s002.pdf]

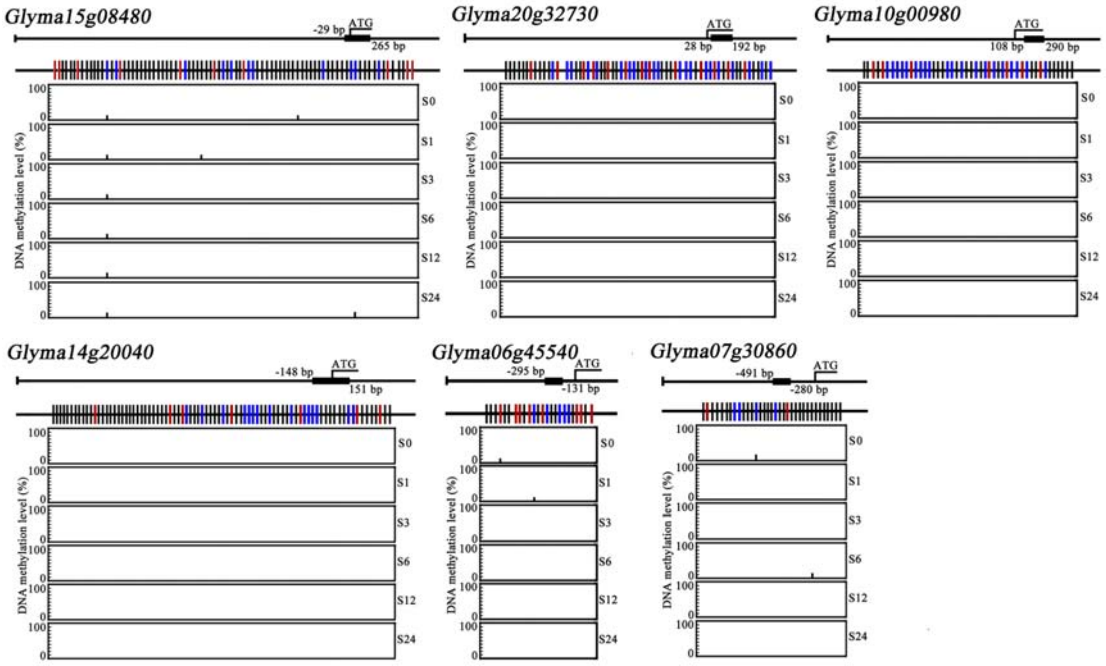

Figure S2. **Genomic bisulphite sequencing of six unmethylated genes in plants not exposed to salinity (S0) and those stressed for 1-24h (S1-S24).** Ten clones of each gene were sequenced. The short thick bars indicate the regions subjected to sequencing, and the long vertical ones show the distribution of CG dinucleotides (black circles), and CNG (triangles) and CNN (unmarked) trinucleotides. The black vertical line represents the proportion of methylated sites. Data represent the mean of three biological replicates.
