# Supplementary material for: The Dynamic Changes of DNA Methylation and Histone Modifications of Salt Responsive Transcription Factor Genes in Soybean
Source: PLoS One. 2012 Jul 18;7(7):e41274. doi: 10.1371/journal.pone.0041274 (PMC3399865; doi:10.1371/journal.pone.0041274)
Supplement: Figure S3 — ChIP analysis to assess the unmethylated gene Glyma20g32730 's H3K9me2, H3K9ac and H3K4me3 content in plants challenged with salinity. The short bars marked “a” indicate regions subjected to genomic bisulfite sequencing (+1 to +393); “I, II and III” indicate the regions subjected to ChIP analysis. (PDF) [file pone.0041274.s003.pdf]

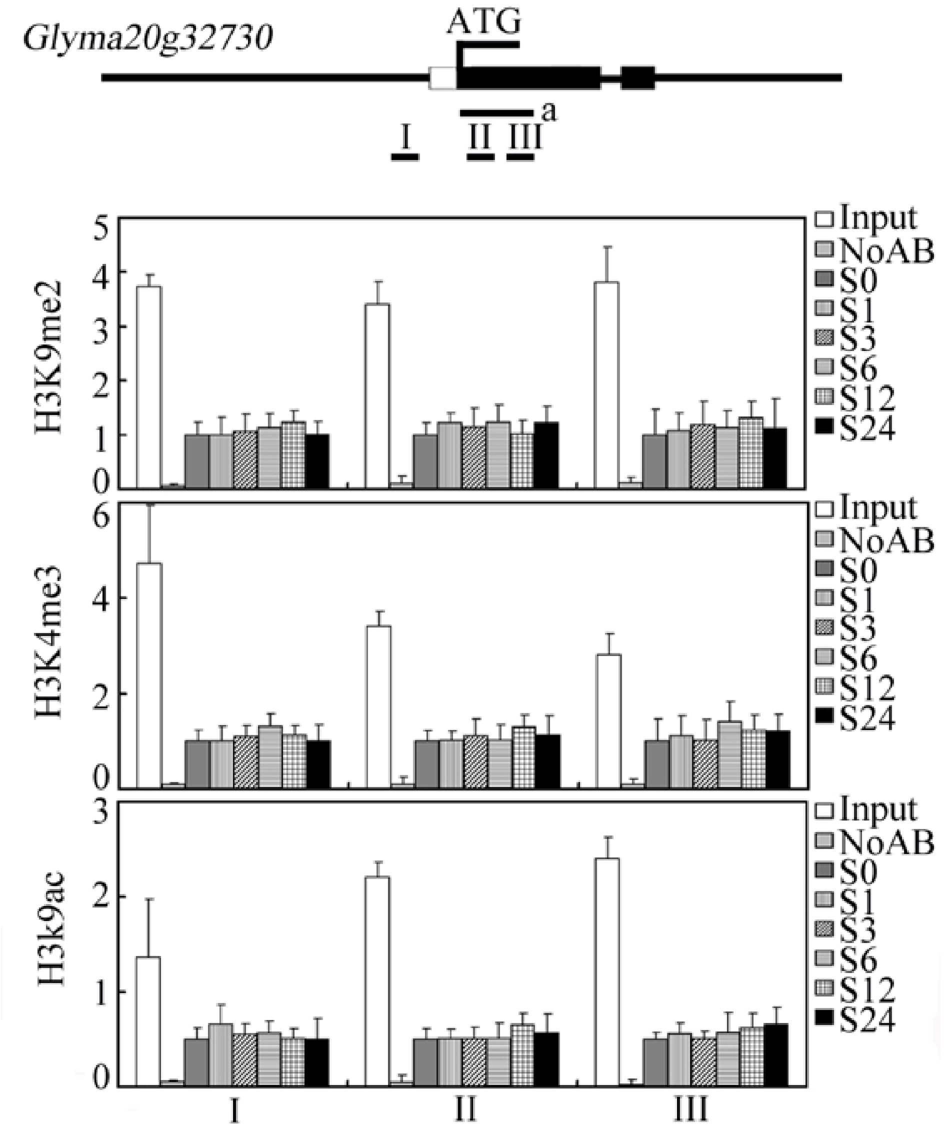

Figure S3. **ChIP analysis to assess the unmethylated gene *Glyma20g32730*'s H3K9me2, H3K9ac and H3K4me3 content in plants challenged with salinity.** The short bars marked "a" indicate regions subjected to genomic bisulfite sequencing (+1 to +393); "I, II and III" indicate the regions subjected to ChIP analysis.
