# Supplementary material for: The Dynamic Changes of DNA Methylation and Histone Modifications of Salt Responsive Transcription Factor Genes in Soybean
Source: PLoS One. 2012 Jul 18;7(7):e41274. doi: 10.1371/journal.pone.0041274 (PMC3399865; doi:10.1371/journal.pone.0041274)
Supplement: Table S1 — The set of TFs identified by microarray analysis to be inducible by salinity stress. (DOC) [file pone.0041274.s004.doc]

**Table S1. The set of transcription factors identified by microarray analysis (fold change >2) as being induced by salinity stress**

| **Probe ID** | **Gene ID** | **Fold change ( Treated VS Control )** |
| --- | --- | --- |
| **The *GmMYBs* that up-regulated under salt stress** | | |
| GmaAffx.42844.1.S1_at | *Glyma02g01300* | 8.111971 |
| GmaAffx.65331.1.A1_at | *Glyma05g31400* | 18.30267 |
| GmaAffx.12474.1.S1_at | *Glyma04g33720* | 5.281899 |
| Gma.18049.1.S1_at | *Glyma05g35050* | 16.345503 |
| GmaAffx.3537.1.S1_at | *Glyma05g37460* | 4.891659 |
| GmaAffx.42044.1.A1_at | *Glyma07g30860* | 6.880071 |
| Gma.18049.2.S1_a_at | *Glyma08g04670* | 16.345503 |
| GmaAffx.3537.1.S1_at | *Glyma11g02400* | 4.891659 |
| Gma.160.1.S1_at | *Glyma12g34650* | 4.9134707 |
| GmaAffx.37381.1.S1_at | *Glyma15g07230* | 7.562643 |
| GmaAffx.494.1.S1_at | *Glyma16g02570* | 2.5205245 |
| GmaAffx.57966.1.S1_at | *Glyma09g24400* | 9.897165 |
| GmaAffx.42059.1.S1_at | *Glyma11g33180* | 14.678792 |
| Gma.14845.1.S1_at | *Glyma06g04010* | 5.711462 |
| Gma.2764.1.S1_at | *Glyma06g45540* | 8.460301 |
| Gma.15808.1.A1_at | *Glyma09g04370* | 3.1897614 |
| **The *GmNACs* that up-regulated under salt stress** | | |
| Gma.8438.1.S1_at | *Glyma06g21020* | 4.539608 |
| GmaAffx.57970.2.S1_at | *Glyma06g38410* | 3.970337 |
| GmaAffx.57970.1.S1_at | *Glyma12g22880* | 20.3571 |
| Gma.895.2.S1_at | *Glyma13g05540* | 3.917188 |
| GmaAffx.13737.1.S1_at | *Glyma14g24220* | 4.2968206 |
| GmaAffx.50811.1.S1_at | *Glyma15g08480* | 2.1294563 |
| Gma.5331.1.S1_a_at | *Glyma06g16440* | 6.892583 |
| Gma.7788.1.A1_at | *Glyma02g07700* | 2.156857 |
| **The *Gmb-ZIPs* that up-regulated under salt stress** | | |
| GmaAffx.62901.1.S1_at | *Glyma03g29820* | 2.8925855 |
| GmaAffx.87012.1.S1_at | *Glyma03g37780* | 2.2705617 |
| GmaAffx.89624.1.A1_s_at | *Glyma04g04170* | 4.196449 |
| Gma.1882.1.S1_at | *Glyma06g08390* | 4.3394612 |
| Gma.8537.1.S1_at | *Glyma08g41450* | 4.339294 |
| Gma.1495.1.S1_at | *Glyma10g08370* | 3.0641787 |
| GmaAffx.42097.1.S1_at | *Glyma12g14130* | 2.608072 |
| Gma.1870.1.S1_s_at | *Glyma14g20040* | 3.3388984 |
| GmaAffx.14279.2.A1_at | *Glyma19g05050* | 2.803964 |
| **The *GmDREBs* that up-regulated under salt stress** | | |
| GmaAffx.16227.1.S1_at | *Glyma20g32730* | 3.7849262 |
| Gma.10100.1.S1_at | *Glyma20g30840* | 3.0465386 |
| GmaAffx.69792.1.S1_s_at | *Glyma16g27950* | 4.0246544 |
| GmaAffx.69508.1.S1_at | *Glyma14g09320* | 2.968299 |
| Gma.15943.1.S1_at | *Glyma11g03900* | 3.942578 |
| GmaAffx.16227.1.S1_at | *Glyma10g34760* | 3.7849262 |
| GmaAffx.5978.1.S1_at | *Glyma10g04210* | 3.248424 |
| GmaAffx.15015.1.S1_at | *Glyma10g00980* | 2.9843888 |
| GmaAffx.2501.2.S1_at | *Glyma09g27180* | 8.770597 |
| GmaAffx.83655.1.S1_at | *Glyma08g14600* | 3.5038126 |
| GmaAffx.36887.1.S1_at | *Glyma07g05240* | 2.968299 |
| GmaAffx.1305.1.A1_at | *Glyma06g06100* | 7.764823 |
| Gma.8372.2.S1_at | *Glyma06g04490* | 2.0336182 |
| Gma.16945.1.A1_at | *Glyma05g03560* | 7.8646255 |
| GmaAffx.84921.1.S1_at | *Glyma02g14490* | 10.748274 |
