# Supplementary material for: The Dynamic Changes of DNA Methylation and Histone Modifications of Salt Responsive Transcription Factor Genes in Soybean
Source: PLoS One. 2012 Jul 18;7(7):e41274. doi: 10.1371/journal.pone.0041274 (PMC3399865; doi:10.1371/journal.pone.0041274)
Supplement: Table S2 — Sequences of primers employed in this research. (DOC) [file pone.0041274.s005.doc]

**Table S2. Sequences of the primers referred to in this research.**

| **Gene ID** | **5’Primer** | **3’Primer** | |
| --- | --- | --- | --- |
| *Glyma02g01300*-RT | GAACATCACCCTTCAAGAACAGCT | TGCCAAAGAGAGTATATGGACCC | |
| *Glyma05g31400*-RT | TATCTAACAAAACCCCTTCTCAAA | CTAACAACCTCAAGACCTCCCTAC | |
| *Glyma05g35050*-RT | CACTGACAGCAGAGAGTTTCAG | ACAATTCATCCATGTTCCACAT | |
| *Glyma05g37460*-RT | ATTAGAAGAGGCAGGTTTACAC | ATATCTCCTTGCTCATCGATAG | |
| *Glyma06g04010*-RT | ATGGAAGCAACCAACAGAAG | AATACCAGGAGGAGCGAGAG | |
| *Glyma06g45540*-RT | CTGGCAAGATGTGGGAAAAG | CAACGGAGGGAGCAAGTAGT | |
| *Glyma07g30860*-RT | AGGCTTGATCTTTTGGACCTATC | CTGTCTCATCCTCAGTGCTGCTG | |
| *Glyma08g04670*-RT | TAAAGCCAGATGTAAAACGGG | TCATATGTGAAGGTGCCAAAG | |
| *Glyma09g04370*-RT | CTAAATGAAAACAAGC CAACC | ACCAATGCCAGTACTCCAC AA | |
| *Glyma09g24400*-RT | ATTCTACTTGGATCCTAGTGG | AAATTGATGGACAGGTTTCTC | |
| *Glyma11g02400*-RT | TCTAAAGCCAGATGTAAAACGG | CAGACAAGGAGATGCATGAACC | |
| *Glyma11g33180*-RT | GGAGGTGGTGTATCTTTGAGT | ATGTTGTTGTTTTGGGTTTGG | |
| *Glyma12g34650*-RT | GCCAGGAAGAACAGATAATGA | TTGAACTTGGGAGAAGAGAAA | |
| *Glyma15g07230*-RT | AAAAATGGATATGGCAATTGGAG | TGAGGTGGAGAAGAGTGAAGAAG | |
| *Glyma16g02570*-RT | GTTGTAGATTGAGATGGATGAACT | TCTGTGGGAAAATAAGAGTGG | |
| *Glyma02g07700*-RT | CGAGTTTGGGGAGAAAGAGTG | CAGATAAGATGGTGCTGAGCAT | |
| *Glyma06g16440*-RT | TGGTACTTCTTTTCTCCTCGG | CCCTCTCGCACCTCACATC | |
| *Glyma06g21020*-RT | CGTTTCGGTTCTTTTGTGTAA | CAGTTTTGGGGAGGTTGTGA | |
| *Glyma06g38410*-RT | GAGTTCCAGAGAGAGACCCTC | TTCCATAGCCAAAAAATTAGC | |
| *Glyma12g22880*-RT | GCCACTGGAACTGACAAAAT | | GGCACAGAAGGGACAAATAA |
| *Glyma12g35000*-RT | CCGACAAGATCATCACCACC | | AGTAACCCCCCTTCACCCA |
| *Glyma13g05540*-RT | AACAGGCAAGGACAGAGAGA | CACGGTAGGGGCAGATTTAG | |
| *Glyma14g24220*-RT | ACACAACTTCACTTACCCCCT | CGACATCTGATTATTCCCGAG | |
| *Glyma15g08480*-RT | ACAGGCCCTCAAAAGCATAC | AACAACCGAGCTGGGAATAA | |
| *Glyma03g29820*-RT | AGATGGCAAGAGGAGAACCA | CAACCACAATGAACTGAGGG | |
| *Glyma03g37780*-RT | CAACAACGCTCGACTACTACAA | TCGGGAATCTCAACGGATAAC | |
| *Glyma04g04170*-RT | GCGTTGGGAATAATAGTGGTT | CAGTTTGTGTTCTTCTTAGGCGT | |
| *Glyma06g08390*-RT | ATCTCGATTACGAGCTTCGCT | TGTTCAGTTGCTGGGGTGTG | |
| *Glyma08g41450*-RT | TGGTGATGTCTTTGTCGCTT | TTCTTCTGACCCTCCCCTGT | |
| *Glyma10g08370*-RT | GATAAGGAAGGAACCAAGCC | TCTACCACCACTGCCAACAC | |
| *Glyma12g14130*-RT | CGGAGGATGAGGATAAAAGC | TGATGGAGATGGGGACTGG | |
| *Glyma14g20040*-RT | GGAAAGAAACGATGTTGCTGA | CTGGTTGTTGATAGGGGTGAG | |
| *Glyma19g05050*-RT | ATGCAAAGCAGGGAAGTAGT | AAAGGGGTTATGAAGGAGGA | |
| *Glyma02g14940*-RT | CCGAGGAACAAAGGAAGAAA | TACCCAGTGGCAGCAGAGTC | |
| *Glyma05g03560*-RT | CCCAACGACAAGTCCACCAG | GATACCTCAGCATCATCCTCCA | |
| *Glyma06g04490*-RT | ATAAGGATGAGGAAGTGGGG | CGTGGCTGGAACTAATGGAG | |
| *Glyma06g06100*-RT | TACAAAGGGGTGAGAAAGCGA | AAGGAAAGGAGAAGGGATACA | |
| *Glyma07g05240*-RT | GAAAGAGAAAATGGGGCAAA | AAACAGAAGGCGATGGAGAT | |
| *Glyma08g14600*-RT | TCCAACCAAATCAAGCTCAAC | CTTCCCAATCAATCTCCACAG | |
| *Glyma09g08330*-RT | TTCCTCTCATCAGCTCCTCCC | TGTTCCAAATAATCAGCCCCA | |
| *Glyma09g27180*-RT | TTCTGATTTGTACCATGTTTCC | CTGCCTTGTATTAGCATTTTTT | |
| *Glyma10g00980*-RT | AGTTCCTCTCATCAGCTCCTCC | GCACATCAACAAGTGTTCCAAA | |
| *Glyma10g04210*-RT | TGTAAATTCCTCTGTTTGTGCA | TTGTTTTTTTTGTTGGTAGCCT | |
| *Glyma10g34760*-RT | TCACCAACTTCAAGCCTCCC | CTCTTCCCTACACCATCCGA | |
| *Glyma11g03900*-RT | GAACCAAACACAACCTCCCT | CATCACTACCCCAGAACCCT | |
| *Glyma14g09320*-RT | CAACAAAGAAAAGAACGGGTA | GTCGGGCTTGAGATTGAGAG | |
| *Glyma16g27950*-RT | AAGGGGGTCAGGTGAGTTTATC | TTCTTCTCTGCCTCTTTTGTGG | |
| *Glyma20g30840*-RT | CGCATTGAGGTTCAGAGGTA | CAGGTGGTGGAGGAGGATAG | |
| *Glyma20g32730*-RT | ACCACAACCAGCAACTCTCT | TGGCTACTGTTCCAATACGA | |
| *GmTublin*-qRT | AACCTCCTCCTCATCGTACT | GACAGCATCAGCCATGTTCA | |
| *Glyma11g02400-*qRT | AAGATTTGAATGAGTGGGTGGAG | GAGGAAGGAAAAGGTGAAAGTGTAT | |
| *Glyma08g41450*-qRT | ACGGCTACTGGTGATGTCTTTG | ACCCTCTTGGTGACTTGTGAAT | |
| *Glyma20g32730*-qRT | GGAGGCAGTGTCACAGACGAAA | GAGAGGCGGCGGAGATAGAT | |
| *Glyma20g30840*-qRT | TCTGCTTCGTATCCTCTGTTTTCTT | ACCCACTACCACCGTCTTCTCC | |
| *Glyma16g27950*-qRT | ACTACAACTGTAACCGCCGTCA | TGTCAAATGTGCCTAGCCAAAC | |
| *GmTublin-*-qRT | GGCAAGATGAGCACCAAGGAA | GTGGAGGACATGGACAAACCC | |
| *Glyma15g08480*-BSP | TGGAATTTGTGTAGTGTTTGAATTT | ACAAACAATCACCATAACCAAATAAT | |
| *Glyma11g02400*-BSP | AATTGTATTTGTTATGATGTAATGA | AAAACCCAAATCTCCATCTAACC | |
| *Glyma07g30860*-BSP | TTTAGAAAGGTATGGGTTATATTTTT | TATCATTTAATAAATTTTCTCAAAATAATT | |
| *Glyma06g45540*-BSP | ATGTAATGTGAAAGTTAAGAAGAAATATAG | TCTATATCTAACCAATCACTTATTTATATA | |
| *Glyma14g20040*-BSP | TGTATATAATAAAGATGTAAAAGTATATGA | AAAATAATTTAATTCCAAACATCCAATATT | |
| *Glyma08g41450*-BSP | TATGGTTTTTTATTTAATTATTTTTATTTT | TCTTTCTTCAATTATTATTATTCCC | |
| *Glyma20g32730*-BSP | ATGGATGGAGGTAGTGTTATAGATGA | TAAAATACATACTAAAAAACTCAACT | |
| *Glyma20g30840*-BSP | TATTAGAGGGGTTTAGGTTTAGGTT | ATAACCAACTCCCAAATAAAAATAC | |
| *Glyma16g27950*-BSP | TTAAAGGGGGTTAGGTGAGTTTATT | AAATCACCCCTATTATACCCTAAAAC | |
| *Glyma10g00980*-BSP | TTAAGAGTAAGTGAATAAGAAGAGGAGTAG | AAATAATCAACCCCAAAATCATAAA | |
| *Glyma11g02400-*ChIP-I | CGTAAGGCACTAAGCGTAAACCA | | AGTAAATCACAGCGAGCGGTTTC |
| *Glyma11g02400-*ChIP*-*II | CATCAGGTTGTAAATGTCGGCTCT | | TCCTGTCTTTTAGTCTTGTTCCCTC |
| *Glyma11g02400-*ChIP*-*III | AACTCATCAGATACATCACCACTCAC | | AAAAAGCAAAAAAGGTTGGTTCA |
| *Glyma16g27950-*ChIP-I | AAACCCACTTTTGATGTGAGCA | | CCATGCTCTGTGCTGTATGCTCTA |
| *Glyma16g27950-*ChIP*-*II | GGTCAGGTGAGTTTATCCGGTT | | TCAACTCTGGATCACCCCTGTT |
| *Glyma16g27950-*ChIP*-*III | CAACAAGGTGAGGTGACAATGC | | CAACCCAAGAGGCTCCAGAAG |
| *Glyma08g41450-*ChIP*-*I | AATGACATTTTTACATCGTCGGTA | | TAATCTGGACTAGGCTAAGACACACT |
| *Glyma08g41450-*ChIP*-*II | ACCACCAATCATTCTCGTTCTT | | AGTATGGGAGTTAGCGGGGTCT |
| *Glyma08g41450-*ChIP*-*III | CACGATCTCTCACAACCAGCCT | | CTTCACCAACTTCACACCTCTCTC |
| *Glyma20g30840-*ChIP*-*I | GCCATTTCTCTTATTCTTACCCATCT | | ACCTACCTCAGTACTCTCTTCCGC |
| *Glyma20g30840-*ChIP*-*II | TGAGGTAGGTTCTGTTCCGAGC | | CAACTCCCAAATGAGGATGCGA |
| *Glyma20g30840-*ChIP*-*III | GCGAGTGGAGGTGTTGCTATTG | | AATGTCCCAAGCCACACTCTCG |
| *Glyma20g32730-*ChIP-I | AGAAGAAAAGAAAAAACTACACCCA | | AACATCCATTAACCTATCTAACGCT |
| *Glyma20g32730-*ChIP-II | GGAGGCAGTGTCACAGACGAAA | | GAGAGGCGGCGGAGATAGAT |
| *Glyma20g32730-*ChIP-III | CTTTGAGGACGTTGGAGGGA | | GAACCGGGTACGGGTAGTTT |
| *GmTublin--*ChIP | GGCAAGATGAGCACCAAGGAA | | GTGGAGGACATGGACAAACCC |
