# Supplementary material for: The Dynamic Changes of DNA Methylation and Histone Modifications of Salt Responsive Transcription Factor Genes in Soybean
Source: PLoS One. 2012 Jul 18;7(7):e41274. doi: 10.1371/journal.pone.0041274 (PMC3399865; doi:10.1371/journal.pone.0041274)
Supplement: Table S3 — Correlation analysis among methylation levels, gene expression and histone modifications of the four TFs during salinity stress. NC: no correlation; *P <0.05, **P <0.01. (DOC) [file pone.0041274.s006.doc]

**Table S3. Correlation analysis**

| ***Glyma11g02400*** | **Methylation** | **mRNA** |  | **Methylation** | **mRNA** |  | **Methylation** | **mRNA** |
| --- | --- | --- | --- | --- | --- | --- | --- | --- |
| H3K9me2-I | 0.78  ** | -0.97  ** | H3K9ac-I | NC | NC | H3K4me3-I | -0.9  * | 0.64  * |
| H3K9me2-II | 0.77  * | -0.96  ** | H3K9ac-II | NC | NC | H3K4me3-II | -0.89  * | 0.76  * |
| H3K9me2-III | NC | NC | H3K9ac-III | NC | NC | H3K4me3-III | -0.83  * | 0.55 |
| Methylation | 1.00 | -0.89  ** | Methylation | 1.00 | -0.89  ** | Methylation | 1.00 | -0.89  ** |
|  |  |  |  |  |  |  |  |  |
| ***Glyma16g27950*** | **Methylation** | **mRNA** |  | **Methylation** | **mRNA** |  | **Methylation** | **mRNA** |
| H3K9me2-I | NC | NC | H3K9ac-I | NC | NC | H3K4me3-I | NC | NC |
| H3K9me2-II | NC | NC | H3K9ac-II | NC | NC | H3K4me3-II | NC | NC |
| H3K9me2-III | NC | NC | H3K9ac-III | NC | NC | H3K4me3-III | NC | NC |
| Methylation | 1.00 | -0.97  ** | Methylation | 1.00 | -0.97  ** | Methylation | 1.00 | -0.97  ** |
|  |  |  |  |  |  |  |  |  |
| ***Glyma20g30840*** | **Methylation** | **mRNA** |  | **Methylation** | **mRNA** |  | **Methylation** | **mRNA** |
| H3K9me2-I | NC | NC | H3K9ac-I | NC | NC | H3K4me3-I | NC | NC |
| H3K9me2-II | 0.83  * | -0.97  ** | H3K9ac-II | -0.82  * | 0.96  ** | H3K4me3-II | -0.83  * | 0.97  ** |
| H3K9me2-III | 0.86  * | -0.89  ** | H3K9ac-III | -0.8  * | 0.96  ** | H3K4me3-III | -0.86  * | 0.89  * |
| Methylation | 1.00 | -0.78  * | Methylation | 1.00 | -0.78  * | Methylation | 1.00 | -0.78  * |
|  |  |  |  |  |  |  |  |  |
| ***Glyma08g41450*** | **Methylation** | **mRNA** |  | **Methylation** | **mRNA** |  | **Methylation** | **mRNA** |
| H3K9me2-I | NC | NC | H3K9ac-I | NC | NC | H3K4me3-I | NC | NC |
| H3K9me2-II | NC | NC | H3K9ac-II | NC | NC | H3K4me3-II | NC | NC |
| H3K9me2-III | NC | -0.67  * | H3K9ac-III | NC | 0.84  * | H3K4me3-III | NC | 0.78  * |
| Methylation | 1.00 | NC | Methylation | 1.00 | NC | Methylation | 1.00 | NC |
