# Supplementary material for: The Dynamic Changes of DNA Methylation and Histone Modifications of Salt Responsive Transcription Factor Genes in Soybean
Source: PLoS One. 2012 Jul 18;7(7):e41274. doi: 10.1371/journal.pone.0041274 (PMC3399865; doi:10.1371/journal.pone.0041274)
Supplement: Figure S1 — Monitoring the non-methylated cytosine to thymine transformation efficiency of the bisulfite sequencing procedure. A fragment of Glyma20g32730 with numerous cytosines was cloned into Dm- E. coli cells and the plasmid was treated with bisulfite. Nine positive clones of this gene from the bisulfite treated DNA were sequenced and compared with the untreated DNA sequence. (PDF) [file pone.0041274.s001.pdf]

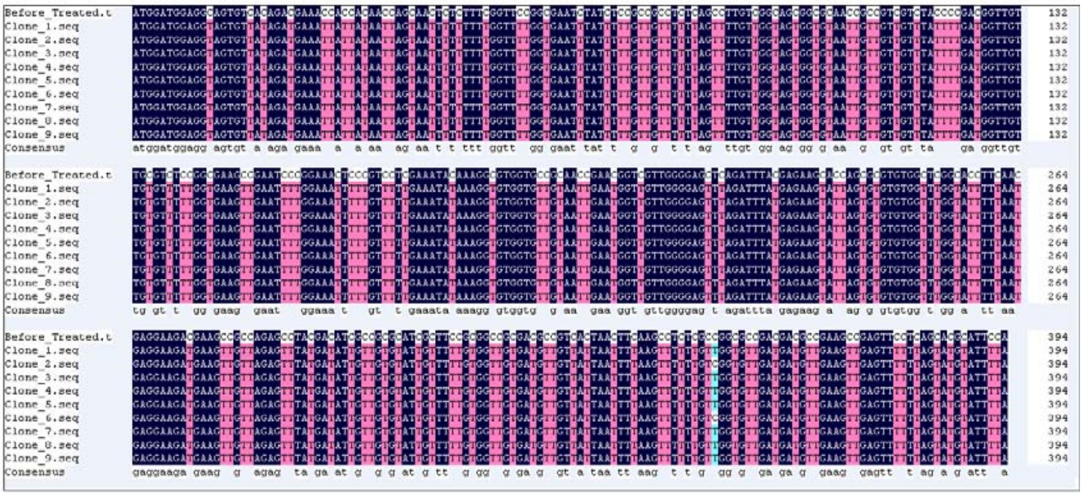

Figure S1. **Monitoring the non-methylated cytosine to thymine transformation efficiency of the bisulfite sequencing procedure.** A fragment of *Glyma20g32730* with numerous cytosines was cloned into Dm- *E. coli* cells and the plasmid was treated with bisulfite. Nine positive clones of this gene from the bisulfite treated DNA were sequenced and compared with the untreated DNA sequence.
